# Impending Death by Chloroform; Resuscitation by Raising the Epiglottis, and Direct Insufflation

**Published:** 1853-04

**Authors:** 


### ARTICLE XXII.

### *Impending Death by Chloroform; Resuscitation by raising the Epiglottis, and direct Insufflation.*

One of Mr. Ricord's pupils has just addressed a letter to this surgeon in *L' Union Médicale*, wherein he states that he succeeded in rescuing from the grave a patient of his (who was being asphyxiated with chloroform,) by following M. Ricord's plan of resuscitation. The lady was having some verrucæ removed from the vulva, &c., and had been narcotized with the handkerchief and pledget of lint impregnated with chloroform within it. She was rather hysterical, and had had a fit when under the influence of chloroform some time previously. In this instance, narcotism was obtained after seven minutes inhalation, and the handkerchief was removed from the mouth from time to time. After the first verrucæ were exercised, sensibility returned; chloroform was given again, and in four minutes the whole operation was finished, but the patient was pronounced pulseless, and the ear applied to the chest could perceive no cardiac sounds. The operator, in great alarm, passed his finger into the posterior part of the patient's mouth, and found the epiglottis covering the entrance of the larynx. By pulling the tongue forward, and pressing the base of the epiglottis, the larynx was freed, and the young surgeon then applied his mouth upon the patient's, and vigorously insufflated the lungs, whilst one of the assistants (there had been two all along) pressed down the parietes of the thorax. This was repeated twice, for five minutes at the time; and the patient, who a few moments before was a mere corpse, gradually recovered, and has done well.

